# Post-Migration Changes in Dietary Patterns and Physical Activity among Adult Foreign Residents in Niigata Prefecture, Japan: A Mixed-Methods Study

**DOI:** 10.3390/nu15163639

**Published:** 2023-08-19

**Authors:** Hansani Madushika Abeywickrama, Mieko Uchiyama, Momoe Sakagami, Aya Saitoh, Tomoe Yokono, Yu Koyama

**Affiliations:** Graduate School of Health Sciences, Niigata University, 2-746 Asahimachi-dori, Chuo-ku, Niigata-shi 951-8518, Niigata, Japan; uchiyama@clg.niigata-u.ac.jp (M.U.); sakagami@clg.niigata-u.ac.jp (M.S.); ayasaitoh8@clg.niigata-u.ac.jp (A.S.); tyokono@clg.niigata-u.ac.jp (T.Y.)

**Keywords:** Japan, immigrants, dietary patterns, physical activity, post-migration changes, mixed-method research

## Abstract

The migrant population of Japan is gradually increasing, but it is not well known how and why diet and physical activity (PA) change post-migration. Therefore, this study used a mixed-method approach to investigate the changes in dietary patterns and PA through a web- and paper-based survey (*n* = 128) and understand the contextual factors for those changes through semi-structured interviews (*n* = 21). Descriptive and thematic analyses of quantitative and qualitative data were conducted, respectively. The majority of survey (57.8%) and interview (66.7%) participants were female, and the mean duration of stay in Japan was 5 and 3.6 years, respectively. The survey revealed an increased consumption of foods attached to Japanese culture, frozen and microwavable food, and a reduced consumption of fruits. We identified environmental (availability, accessibility, and affordability of foods; food safety and diet-related information; and climate), individual (living status; post-migration lifestyle; and food preferences and limitations), or socio-cultural (relationships with Japanese people; cultural differences; and religious influences) factors that impact diet changes. Language proficiency and the duration of stay shape dietary behaviors. Determinants of PA changes were climate, lifestyle, and the influence of Japanese culture. In conclusion, immigrants in Japan experience post-migration diet and PA changes, and this study adds knowledge about how and why such changes occur.

## 1. Introduction

Migrants are considered a vulnerable population group for nutrition- and lifestyle-related chronic diseases due to the shift in diet patterns and lifestyle with the change in the environment [1]. Previous studies have reported a higher body mass index and waist circumference in European Asians compared to counterparts with similar cultural and genetic backgrounds in their country of origin [2,3,4]. Significantly high prevalence rates of diabetes among ethnic minority groups from Turkey, Vietnam, Sri Lanka, and Pakistan compared to Norwegians [5] and among British South Asians than in the indigenous population have also been reported [6]. The process by which migrants adopt the dietary practices of the host country is referred to as ‘dietary acculturation’ and can be influenced by a variety of demographic, economic, social, and cultural factors [7]. Dietary acculturation is not linear but rather a complex, dynamic, and multidimensional process where immigrants retain or find new ways to use foods from their home country (from here on referred to as traditional foods), exclude traditional foods, and/or adopt the diet of the host country [8]. Satia-Abouta et al. [8] proposed that complex and dynamic relationships of socioeconomic, demographic, and cultural factors predict the extent to which immigrants change their diet-related attitudes and beliefs, taste preferences, and food purchasing and preparation methods, ultimately leading to changes in dietary patterns.

While several studies have been conducted in European and North American countries to assess the post-migration changes in diet and lifestyle among immigrant communities [2,3,4,5,6,9,10,11], not many studies have been conducted in Asian countries. Particularly, East Asian countries such as Japan, China, and South Korea have become popular destinations among migrants in recent years. A recent study has reported changes in dietary patterns among Central Asian immigrant workers in South Korea [12]. In 2020, approximately 2.89 million foreign residents were registered in Japan, making up about 2.3% of the population [13]. More than 80% of foreign residents in Japan are of Asian origin, mainly from China, Vietnam, South Korea, and the Philippines. South Americans account for about 10% of the immigrant population, while those from North America and Europe represent less than 5%. The proportion of Africans and Oceanians among the immigrant population is less than 1%, according to the most recent statistics available [13]. As companies in Japan face challenges with labor shortages due to the country’s shrinking workforce and aging population, there is a greater demand for foreign workers. As a result, the number of foreign residents in Japan has increased substantially over recent years, and the long-stay migrant labor is expected to boost.

Japan is widely recognized as a healthy country, with the highest (healthy) life expectancy at birth in the world [14] and the lowest rate of obesity among adults in OECD countries [15]. The Japanese dietary pattern is characterized by high intakes of fish and seafood, green and yellow vegetables, Japanese pickles, green tea, beans, and bean products. It is well-recognized to be associated with a lower risk of disability and death [16]. Japanese food culture is also rich in noodle-based cuisines such as ramen, soba (buckwheat noodles), and udon (thick Japanese noodles). According to a national survey conducted in 2022, 20.2% (estimated 21.30 million) of the adult Japanese population were active participants in sports and physical activities, with about 30% ‘strolling’ or ‘walking’, while about 15% were engaged in ‘calisthenics and light exercises’ and ‘weight training’ [17]. As the adaptation to a less healthy American diet leads to increased chronic diseases among migrants in the United States [10,11], it would be intriguing to assess the changes in dietary patterns among foreign residents in Japan. It was also our interest to investigate how the PA of migrants changes when they navigate their life in Japan. A better understanding of these post-migration changes and associated factors among migrants is important as they can be associated with various health consequences and may have implications for the planning and implementation of support programs for foreign residents. To our knowledge, studies about the dietary and lifestyle transition of migrants in Japan are scarce. The aim of the present study is to explore post-migration changes in dietary patterns and physical activity (PA) and contextual factors among foreign residents in Japan.

## 2. Materials and Methods

### 2.1. Study Design and Sample

A mixed-methods explanatory sequential design was adopted, combing a cross-sectional survey and semi-structured interviews, and conducted from July 2022 to April 2023 in Niigata prefecture, Japan. In this approach, quantitative data collection was followed by qualitative data collection with the intention of reporting the self-reported changes in dietary patterns and PA after migration (quantitative) and contextualizing how and why the changes occurred (qualitative) [18]. The study population included foreign residents who were 18 years old or over, able to read and speak in English or Japanese, and resided in Niigata prefecture for more than 6 months to ensure that they had settled in the environment and could offer meaningful responses. Those who were pregnant or had a medical condition that required a prescribed therapeutic diet were excluded, as the perceived changes might be primarily associated with their condition but not with migration.

### 2.2. Participant Recruitment

Survey participants were recruited through flyers, social media, and personal outreach through community contacts of the research team. Survey respondents were informed about the interview phase of the study at the end of the questionnaire and requested to provide a contact method (e-mail, telephone number, and/or an address) if they wished to participate in the interview. A maximum of three attempts were made to contact all the survey respondents who provided a contact method. Those who responded expressing their willingness to participate in the interview were provided with an information sheet and recruited upon their consent. Interviewees were compensated for their participation.

### 2.3. Data Collection

A self-administered questionnaire in both paper-based and web-based formats was used for data collection. Japanese and English versions of the questionnaire were available and pre-tested using 10 foreign residents. The questionnaire was designed referring to the model of dietary acculturation proposed by Satia-Abouta et al. [8] and consisted of questions on the following:

1. Demographic, socioeconomic, and cultural data (14 questions on sex, age, country of origin, religious affiliation, years in Japan, education, income, employment, household composition, fluency in the Japanese language, and dietary habits)
2. Perceived changes in portion size, food consumption (53 food items), preparation (7 items), and dietary behaviors since immigration (14 items)
3. Factors associated with the procurement and preparation of traditional foods (availability, accessibility, and affordability of traditional foods/restaurants) (4 questions)
4. Perceived changes in PA behavior since immigration (3 questions)

All interviews were conducted by one researcher (H.M.A.) in person or via an online video communication platform (Zoom Video Communications Inc. platform, San Jose, CA, USA) according to the participant’s preference. A semi-structured interview guide with open-ended questions on major changes in diet and PA since immigration to Japan, the timing of and reasons for those changes, cultural differences in the food practices and lifestyle between the home country and Japan, and the availability/affordability and accessibility of traditional foods were used. All the interviews were conducted in English and video and/or audio recorded with the interviewees’ consent. The interviews were 30 min to 1 h in length.

### 2.4. Ethical Considerations

Ethical clearance was obtained from the Ethical Review Committee on research involving human subjects at Niigata University, Japan (No. 2022-0005). Each survey participant received a detailed explanation about the study at the beginning of the questionnaire, and upon carefully reading the information, they were asked to confirm their consent in writing or electronically. Verbal consent was obtained at the beginning of the interview. The consent was sought to report the selected quotes from each interview participant, and all data were anonymized during reporting.

### 2.5. Data Analysis

The analysis of quantitative data was conducted using SPSS (version 26.0, SPSS Inc., Chicago, IL, USA). Descriptives were presented as frequencies (*n*) and proportions (%) for categorical variables and mean values and standard deviations (SD) for continuous variables. The chi-square test of independence was performed to assess the relationship between categorical variables. Differences were regarded as significant at a two-tailed *p*-value *<* 0.05. The interviews were transcribed verbatim, and a thematic analysis of data was conducted following 6 steps: (i) familiarizing with the whole dataset; (ii) creating codes; (iii) searching for themes among the codes; (iv) reviewing themes; (v) defining and narrowing themes; and (vi) reporting/explanation [19]. The analysis was inductive. Additionally, open comments from survey respondents were also considered in the qualitative analysis. Initial codes were generated independently by two researchers (H.M.A. and A.S.), and from these, codes were categorized and merged into themes that reflect how and why shifts in dietary patterns and PA occurred. The two authors continued coding and generating themes until thematic saturation was achieved. Upon reaching a consensus on their analysis, the two authors reviewed and refined themes and sub-themes with the study team. After several stages of revisions and discussions, themes were grouped until all the data fit well into three categories. An agreement among research team members on the final categories and themes was sought to achieve the credibility of the qualitative analysis. Further, study participants were asked to review the accuracy of transcribed data and confirm the resonance with their experiences. Thematic maps were created for each category to illustrate how and why changes occurred.

## 3. Results

The findings from the mixed-method data set were interpreted using a contiguous approach. We thus report quantitative and qualitative data independently [20].

### 3.1. Sample Characteristics

**Figure 1:**
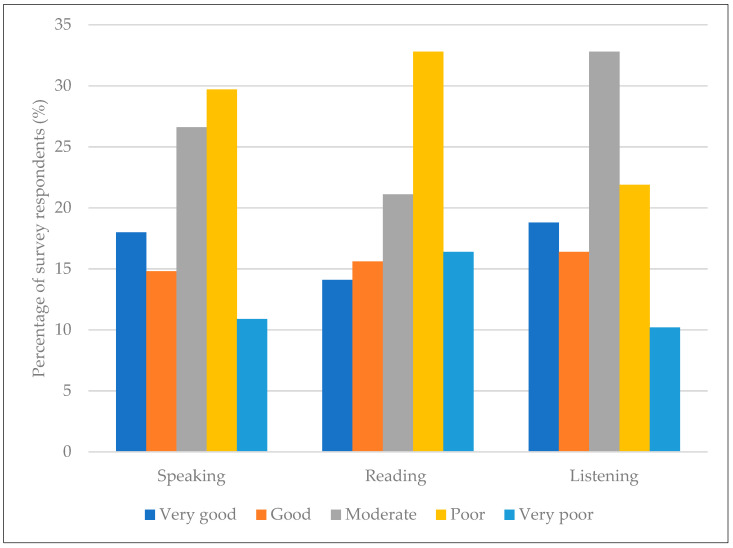
Japanese language proficiency of survey participants (*N* = 128).

In total, 128 survey responses were received, and the socio-demographic characteristics of the respondents are presented in Table 1. The total sample consisted of 54 (42%) male and 74 (58%) female participants. The mean age of the participants was 32.6 (8.4), ranging from 18 to 68 years. The majority of the participants were from Asia (see Table S1.1 for representation from each country) and were students (*n* = 85). Most participants were unmarried, living alone, and had a monthly income of 100,000–200,000 ¥. About 42% of participants were married or living together, and among them, 35 (68.6%) had a partner of the same nationality, while the partner was a Japanese national in 14 (27.5%) of them. The mean length of residency in Japan was 5 years (SD—6.3, range = 0.5–40 years). Most respondents had no particular dietary habits and ate traditional foods at least once a week (63.3%). There were no significant associations between the frequency of traditional food consumption and demographic factors. While a significant association was found between the absence of particular dietary habits/restrictions and the lower frequency (<4 times a week) of traditional food intake (χ^2^ (1, *N* = 128) = 5.222, *p* = 0.022), traditional dishes were consumed more frequently (≥4 times a week) by those with restricted beef intake (χ^2^ (1, *N* = 128) = 7.991, *p* = 0.007; see Table S1.2). In general, fluency in the Japanese language was very poor to moderate (Figure 1).

A total of 21 interviews were conducted, and the characteristics of the interview participants are summarized in Table 1. Interviewees ranged in age from 24 to 55, while the majority were female and university students, with a monthly income of 100,000–200,000 ¥. Two of the interviewees were married to a Japanese national. Many interviewees noted that they were consuming traditional foods occasionally or rarely.

### 3.2. Self-Reported Changes in Consumption of Foods/Food Groups

About 47% (*n* = 60) of the respondents reported changes in portion size after coming to Japan; among them, 58% (*n* = 35) mentioned that they eat much less than in their home country. Table 2 summarizes the list of foods with more than 50% of total change (eat much less or more compared to their home country). More than half of the respondents reported an increased consumption of noodles, raw fish, seaweed, seafood, green tea, soy sauce, and miso and reduced intake of fruits (See Table S1.3 for the detailed food list). A statistically significant relationship was found between low fruit consumption and low monthly income (χ^2^ (1, *N* = 128) = 25.467, *p* = 0.002; See Table S1.4).

### 3.3. Self-Reported Changes in Food Preparation Methods and Dietary Behaviors

Food preparation methods and dietary behaviors for which more than 50% of respondents noted some sort of change are presented in Table 3 (see Table S1.5 for the detailed list). Increased practices of microwaving (76.5%) and using frozen foods (64.7%) were more prominent among students (see Table S1.6).

### 3.4. Factors Associated with the Procurement and Preparation of Traditional Foods

About half of the respondents reported that the availability of traditional food ingredients and restaurants is limited, while about 75% mentioned the higher prices of ingredients (Table 4). The availability of traditional food ingredients was associated with the higher frequency of traditional food consumption (χ^2^ (1, *N* = 128) = 4.001, *p* = 0.045; see Table S1.7). Online orders within Japan and international shops were the most common methods of purchasing traditional ingredients. The other methods of procuring traditional food ingredients noted by respondents were driving to international shops in other prefectures and asking friends who visit the home country to bring them.

### 3.5. Perceived Changes in Physical Activity

Nearly 73% reported changes in PA levels since immigration, while 55 (43%) mentioned increased PA levels. These changes were more evident in terms of walking, stair climbing, household chores, and cycling (Table 5). One hundred and four (81.3%), fifteen (11.7%), and nine (7.0) respondents described their lifestyle pattern as mostly standing or sitting, mostly walking or stair climbing, and heavy work, respectively.

### 3.6. Factors Influencing Post-Migration Dietary Changes: Qualitative Analysis

The thematic analysis of qualitative data identified 10 themes (factors) belonging to three categories (environmental, individual, and socio-cultural). Sub-themes were identified for each factor with regard to how and why dietary changes occurred. These factors and sub-themes are reported below with illustrative quotes (selected illustrative quotes for each sub-theme are summarized in Table S2.1). Thematic maps of qualitative findings are reported in Supplementary File S3.

#### 3.6.1. Environmental Factors

Figure S1 illustrates the thematic map of the environmental factors influencing post-migration dietary changes.

Affordability of Foods and Ingredients

This factor consists of two sub-themes: (1) higher or lower prices of foods/meals and (2) adaptive purchasing and dietary behaviors. Many participants found that the cost of certain foods, mainly fruits, vegetables, meats, and traditional spices, are high compared to their home countries, while some Japanese foods like natto (fermented soybeans), tofu, etc., are more affordable to incorporate into their diet. The higher prices have made participants change their dietary behaviors by opting for cheaper foods, buying smaller quantities, or when the prices are lower. The cost of eating outside has led the participants to prepare their own meals, which they did not carry out back in their home countries.

The duration of stay in Japan and employment status shapes purchasing behavior. Recent immigrants tend to compare the prices between the host country and their home country when buying food and ingredients.

“*I just think that everything is more expensive because I got here several months before. Fruits and vegetables here are very expensive. So, when I buy something, I still compare it with the Rupiah (Indonesian Currency).*”

(Interview #12, Female, Indonesia)

Further, the purchasing and dietary practices adopted upon arrival in Japan can become a habit even though the circumstances change later.

“*I eat a lot of chicken here in Japan but not a lot of meat. I mean, like beef. I think it is because of the price. Because first, when I came here, I was just a student. So, at that moment, I saw that the meat was very expensive. Now, I am working and I can afford it, but maybe it became some kind of habit for me.*”

(Interview #13, Female, Kazakhstan)

##### Availability and Accessibility of Foods and Ingredients

This theme is composed of the following sub-themes: (1) a limited/wide availability or accessibility of foods or ingredients, and (2) adaptive purchasing and dietary behaviors. Participants expressed that while some ingredients/foods are less or not available (traditional spices, vegetarian food, halal food, and foreign food restaurants), some are difficult to procure in the same quantities (beef) or qualities as in their country of origin (traditional flavors). Difficulties in accessing traditional foods and ingredients were also discussed by many participants. On the other hand, participants reported that some foods and ingredients are widely available (tofu, mushrooms, and frozen foods) and easily accessible (convenience stores and bakeshops) in Japan compared to their home countries.

In response to the limited availability and accessibility, immigrants have adopted measures such as finding products that can be used as substitutions for traditional ingredients, fusing Japanese and traditional cuisines, ignoring the non-availability of ingredients that do not affect the flavor of traditional dishes significantly, and giving up the preparation of some traditional dishes. Due to the lack of stores or restaurants in Niigata that cater to the international community, immigrants have adopted measures such as driving to other cities where international stores are available, ordering online, or acquiring the skills to prepare and cook traditional/international dishes independently.

The duration of stay in Japan and fluency with the host language affect the accessibility of food.

“*After a lot of searching when I first got here, I found the places where I could buy what I needed in Niigata. Like I know exactly where to go if I want pure ground beef rather than mixed pork and beef. There are certain places that sell that. So, when I am preparing local dishes sometimes, I have to get out on the car and go to about 4 to 5 different places to find everything I need. But I know exactly where to go now.*”

(Interview #5, Male, USA)

“*I try to learn some Japanese foods, but my main problem is the language. I mean, when I go to the supermarket, I cannot distinguish well the ingredients. For example, if I find a recipe in English on the internet for Japanese food, I am like, ‘ahh, okay,’ and then I go to the supermarket, and it is difficult for me to find some things. For example, for soy sauce, there are different varieties. So, I cannot understand which one I need.*”

(Interview #1, Female, Chile)

##### Food Safety and Related Information

Participants discussed (1) the perceived safety/unsafety of foods and ingredients in Japan compared to their home countries and (2) the lack of food-related information or knowledge. In contrast to the participants who described foods in Japan as safe and clean, some expressed their fear of buying products or preparing foods unavailable in their home countries due to a lack of related information or knowledge.

##### Climate in Niigata

The cold weather in the Niigata prefecture was mentioned as a factor that affected the changes in food intake (an increased rice amount and drinking tea and coffee) and food preparation methods by some participants.

“*The weather in Japan, it is very cold for me. The region I live in Indonesia is quite hot, and it’s a tropical country. I think it is easy for me to get hungry here, and I want to eat rice more than I’m in Indonesia. Also, it is so cold in my apartment, so I have to cook something easy and quick, so I just fry it.*”

(Interview #17, Female, Indonesia)

#### 3.6.2. Individual Factors

Figure S2 illustrates the thematic map of the individual factors influencing post-migration dietary changes.

##### Family Structure/Living Status

The sub-themes include (1) the burden of preparing complicated or time-consuming meals for one person, (2) Japanese–foreign households, (3) the newfound responsibility of procuring and preparing foods, and (4) changes in meal-associated family traditions. While those who were living alone felt that preparing a full-course meal or time-consuming meal is a burden, the dietary habits of those with a Japanese spouse/partner reflected the two cultures (traditional foods of the foreign resident and Japanese-style meals).

“*I got divorced recently, but when we were living together, we did do the whole, make the broth for two and half hours and do the soup. I don’t have that kind of time right now. When cooking for people, I do a little bit more special, but for myself, no need of it.*”

(Interview #10, Female, Russia)

Some participants found themselves in a situation where they have to bear the responsibility of procuring and preparing food post-migration. Some interviewees mentioned that they no longer follow certain meal-associated family traditions in Japan, such as tea time.

##### Food Preferences and Limitations

The sub-themes of (1) preference for traditional foods/tastes, (2) newfound preference for Japanese foods/tastes, (3) willingness to try new foods, (4) ingrained habits and beliefs, and (5) health-related factors were identified under this theme. Interviewees express their preference for traditional foods/tastes in terms of missing traditional foods, avoiding Japanese products with flavors different from traditional products, and choosing foods with similar tastes or qualities to traditional foods.

“*I prefer Chinese food because it’s quite similar to Indonesian food. So, I prefer to go to Chinese restaurants than Japanese restaurants.*”

(Interview #17, Female, Indonesia)

According to participants’ narrations, the newfound preference for Japanese foods/tastes and willingness to try new foods were catalysts for embracing Japanese foods, which led to an increased intake of Japanese foods, preparation of Japanese foods by themselves, and fusion of traditional and Japanese cuisines.

“*I like to explore a lot, and I like to be creative, so sometimes I try to cook something, kinda Mexican style but with Japanese ingredients, I like to try some new stuff, and there are no restrictions. Zero.*”

(Interview #18, Male, Mexico)

Ingrained habits and beliefs and health-related factors influenced the post-migration diet changes by limiting or increasing the intake of some Japanese foods, while some immigrants described that their dietary habits are quite similar to those of their home country.

##### Post-Migration Lifestyle

Time restrictions and a busy lifestyle came up as factors of post-migration dietary changes, especially among students. A lack of time resulted in opting for simple foods that do not require much preparation time, bulk food preparation and storing, choosing foods that do not spoil quickly, missing meals (especially breakfast), or a fusion of traditional and Japanese cuisines.

“*I am having Russian style, like lots of vegetable soups, but the bases and broth…it’s like Miso because I don’t have a lot of time to make the broth, I’m just like, Miso is fine. It’s a kind of combing of foods.*”

(Interview #10, Female, Russia)

In addition, the difference between pre- and post-migration lifestyles has led some participants to start preparing and procuring food on their own.

#### 3.6.3. Socio-Cultural Factors

Figure S3 illustrates the thematic map of the socio-cultural factors influencing post-migration dietary changes.

##### Contrast between Traditional and Japanese Food Cultures

The sub-themes identified were (1) the influence of the characteristic features, (2) the positive effects, and (3) the problems of the Japanese food culture. According to the narrations of interviewees, they have incorporated characteristic features of the Japanese food culture into their post-migration diet, such as raw fish and a variety of root vegetables, tea with meals, and one-bowl meals in contrast to set meals in traditional cuisines. Participants described positive features of the Japanese food culture, including light meals, fresh foods, and low temptations for unhealthy foods, compared to their traditional food culture. However, light meals have resulted in an increased meal frequency as getting hungrier is faster than with their traditional diets. A lack of vegan or vegetarian options, additives, and higher sodium and sugar contents emerged as problems associated with the Japanese food culture.

Notions regarding Japanese culture among other nationals have resulted in dietary changes even before moving to Japan.

“*Actually, before coming to Japan, my professors [in India] used to say that if you go to Japan, you should be non-vegetarian. Because, when the professors [in Japan] offer some food, you should not say ‘no.’ It is like a…kind of disrespect. And then I started to eat non-vegetarian food, like 6 or 7 months before coming to Japan.*”

(Interview #2, Male, India)

##### Relationships with Japanese People

Some participants described how having close connections with Japanese people broadened their view of authentic Japanese foods and provided opportunities to explore and learn about Japanese cuisines. Further, Japanese language proficiency shapes the relationships with locals.

“*Since I use a lot of Japanese in daily life, I have a lot of opportunities to get along with Japanese in general, like connect deeply. And a lot of elders share some secrets or give us some food. So, I think for me, the language opened a lot of doors and a lot of opportunities to try new stuff and get to know a lot of things.*”

(Interview #18, Male, Mexico)

Participants also highlighted how the COVID-19 pandemic impacted their relationships with locals and their diet behavior subsequently.

##### Religious Influence

Interview narrations revealed the influence of religion on the post-migration diet, which resulted in limiting eating out, checking the ingredients of foods before purchasing, and preparing their own meals.

### 3.7. Factors Influencing Changes in PA since Migration: Qualitative Analysis

Factors influencing PA changes were weather, post-migration lifestyle, and characteristics of Japanese culture. These factors and sub-themes are reported with selected illustrative quotes in Table S2.2. Figure S4 illustrates the thematic map of the factors influencing post-migration PA changes. The cold winter in the study area limits going out and engaging in PA or opting for indoor PA, while certain characteristics of Japanese culture, such as a health-conscious lifestyle (being in good shape, riding bicycles, and jogging), small apartment rooms, and perceived safety, drives PA behaviors. Although most participants expressed that they are walking more compared to their home country, some participants reported that changes in their PA are situational and not Japan-specific.

“*There’s a change in physical activity, more physical activity in general. I did start and also stop doing various sports while here [in Japan] as a means of meeting people. So, more physical activity because I am going to places. Even if I lived in another country, this is the same, and this is not something Japan-specific. But I would say riding a bicycle is a Japan-specific thing, and starting sports is not Japan-specific, it’s just a way of meeting people and having a community without too much effort. I got a car last year, so the physical activity kind of dropped, especially in this kind of weather*.”

(Interview #10, Female, Russia)

### 3.8. Summary of Changes and Adaptive Behaviors of Diet and PA after Migration

Table 6 summarizes how and why the post-migration changes and adaptive behaviors occurred.

## 4. Discussion

This study sought to explore the post-migration changes in dietary patterns and PA levels of foreign residents living in Niigata prefecture, Japan, and the factors affecting such changes. To our knowledge, this study presents the first insight into diet and PA changes among migrants in Japan without limiting them to a nationality or a region. The findings of this study will add to the scarce body of evidence on immigrant health in Japan. The survey respondents perceived an increased consumption of foods associated with the Japanese traditional diet and the use of frozen and microwavable food, and the decreased consumption of fruit. According to participants’ narratives, different factors influence their eating behavior and PA, which can be environmental (availability, accessibility, and affordability of foods; food safety and a lack of food-related information or knowledge; and climate), individual (living status; post-migration lifestyle; and food preferences and limitations), or socio-cultural (cultural differences; relationships with Japanese people; and religious influences). Certain factors related to immigration and settlement, such as the duration of stay and Japanese language proficiency emerged as factors that shape dietary changes. Factors associated with PA changes were weather, post-migration lifestyle, and characteristics of the host culture.

During adaptation to Japanese food culture, an increased consumption of certain feature foods and ingredients in Japanese cuisines, such as noodles, raw fish, seaweed, seafood, green tea, soy sauce, and miso, is more evident. A recent study conducted in South Korea has reported an increased consumption of vegetables, kimchi, rice, and fisheries’ produce, which are common in the Korean food culture, among Central Asian migrant workers [12]. The dietary changes of migrants from different regions are inconsistent owing to the different food cultures in each part. For instance, all the participants from the Americas reported that they are eating more noodles in Japan compared to their home countries, while there was no significant change in the noodle intake of many respondents from East Asia (Table S1.8). Noodle-based cuisines are readily available in Japan as prepackaged meals in supermarkets and convenience stores in addition to local restaurants and food stalls. Further, cup noodles are quite popular as a cheap and easy-to-make meal. According to interview narrations, the preparation of noodle dishes was simple compared to other Japanese food. For instance, one participant mentioned,

“*I sometimes cook some Japanese food like ‘yaki soba’ or ‘yaki udon’ (fried noodles), something simple like that. Not complicated.*”

(Interview #14, Female, Türkiye)

Niigata prefecture is located along the coast of the Sea of Japan and is well-known for its fresh marine produce. The interview participants from land-locked countries and regions described that seafood is fresh, more affordable, and accessible in Niigata prefecture. Most participants from South and Southeast Asia reported an increased consumption of green tea, as black tea culture is common in these regions (Table S1.9). An increased use of frozen and microwavable foods was observed, attributing to the larger representation of international students in the study sample. Interview narrations further revealed that the availability and easy accessibility of frozen foods also affected the increased consumption. Due to the abundance of fresh produce throughout the year and negative public perception, frozen and chilled foods’ usage is limited in South and Southeast Asian countries (Table S1.4).

Satia et al. [8] described the cumulative effect of sociodemographic and cultural factors, changes in diet-related environmental and psycho-social factors, and the level of exposure to the host culture on the dietary intake of immigrants. Our findings are also consistent with a previous study, which reported the availability, accessibility, and affordability of dietary products, and time management, as key determinants of change in dietary behaviors [21] among Australian residents of Sub-Saharan African ancestry. A lack of family support, reduced access to traditional food ingredients, and curiosity regarding new foods in the host country were found as determinants of the dietary changes among recent Chinese migrants in Australia [22]. Many interviewees discussed that imported ingredients or products from their home countries are considerably costlier due to import taxes or limited availability. In addition to the high cost or less availability, they highlighted the lack of diversity in fruits, vegetables, and certain meats in Japan compared to their home countries. The cross-sectional study results align with these findings, as most respondents reported a limited availability and high cost of traditional food products as well as a reduced intake of fruits. Survey results further showed an association between monthly income and fruit intake. Particularly, a reduced intake of fruits and vegetables is of concern as it is a risk factor for chronic diseases [23]. The combined effect of living alone and a busy lifestyle on reduced fruit and vegetable consumption also emerged as they opt for foods that do not perish quickly and do not require going to the market frequently. Due to the interplay between the availability, affordability, and accessibility of food ingredients, the migrants are inclined to explore cheaper or widely available alternatives and omit or substitute the ingredients in traditional food recipes [24].

The changes in family structures influenced post-migration eating behavior. Most of the participants in the current study were foreign students who were living alone. The interview narrations revealed that most students lived with their families before moving to Japan, and they were not solely responsible for their diet. Therefore, the newfound responsibility to purchase and prepare food on their own, combined with time restrictions, has made the participants opt for more convenient and affordable options. Characteristics of the study area emerged as the factors affecting the dietary changes. For instance, the climate of Niigata prefecture is characterized by long cold winters with heavy snowfall and strong winds, which is different from the climate of the southern parts of Japan. Further, interviewees narrated that while foreign food restaurants and shops that offer international food are limited in Niigata prefecture, those are widely available in large metropolitan areas with a high immigrant density, such as Tokyo. Therefore, they have found ways to procure traditional ingredients by ordering through online shops or driving to shops in other prefectures. Previous studies have also reported that urban areas with a high proportion of immigrants had better access to ethnic foods and stores that cater to their food preferences [25,26]. Those who described themselves as ‘food explorers’ with the willingness to try new foods endorsed the Japanese food culture by incorporating many readily available and affordable Japanese foods, such as tofu, natto (fermented soybeans), miso, etc., into their regular diet or mixing the Japanese and traditional ingredients to modify the ethnic recipes. Survey findings of the current study showed that the frequency of traditional food consumption is associated with individual dietary habits and the availability of traditional ingredients. Previous studies have reported that accessibility and individual food preferences affect the overall well-being or satisfaction of migrants in their new environment [26].

As highlighted by the interview narrations, the duration of stay and Japanese language proficiency shape dietary changes. As the duration of their stay increases, immigrants are exposed to mainstream food culture and become more open to trying local foods and adapting to the local diet [8]. In addition, longer stays provide opportunities for a deeper exploration of food markets and methods to procure traditional ingredients, which can lead to adopting bicultural eating habits. The relation between changes in dietary habits and the length of stay in the host county was reported in previous studies [8,27,28]. Language proficiency provides migrants the opportunities to form deeper connections with the locals. Such relationships help gain insight into Japanese cuisines, ingredients, and authentic tastes [8,26]. The use of English to communicate is limited among local residents in Japan, who can introduce migrants to local dishes and ingredients, local food establishments that may not be easily accessible to migrants, and cultural food practices, such as seasonal eating and dining customs. A good command of the language also facilitates reading labels and understanding nutritional information, effectively communicating food preferences and restrictions to local vendors and restaurants, and asking questions about ingredients, cooking methods, etc., which can be important for making food choices. Therefore, fluency in the Japanese language plays a crucial role in accessing and understanding local food culture and enables the migrants to make informed choices about their diet.

Japanese locals are known for their health-conscious and relatively active lifestyle. According to the Global Health Observatory figures in 2016, 65% of adult Japanese people met the recommended level of PA by the World Health Organization [29]. Interview narrations of the current study suggested that the physical and cultural environment in Japan is supportive of PA and acts as a catalyst for increased PA levels. In particular, Niigata prefecture has a PA-supportive physical environment equipped with jogging, bicycle, and skiing trails, leisure parks, mountains, and surfing spots. The culture in Japan values staying active, sports, and engaging in outdoor recreation, which in combination with perceived safety, might encourage migrants to be physically active. Some interviewees acknowledged that increased PA levels are not specifically due to the Japanese influence but rather global trends. The majority of the immigrants in the current study described their daily life as ‘mostly sitting and standing,’ and were engaged in some form of unstructured PA (walking, climbing stairs, and cycling) rather than regular sports. These findings are likely to be associated with the composition of the survey population, which consisted of 65% of international students. In spite of the PA-supportive environment in Niigata prefecture, its climate was recognized as a barrier to outdoor PA. A recent study conducted in Norway described the changes in PA habits among Italian immigrants. They reported a perceived increase in PA levels after moving to Norway and that Italian immigrants in Norway are more physically active than their counterparts in Italy [30].

### 4.1. Strengths and Limitations

A key strength of this study was the mixed-method approach, which combined the qualitative data to contextualize the quantitative results. Another strength was the diversity in the sample population, which consisted of participants from different regions of the world and socio-demographic backgrounds. However, caution should be taken when generalizing the findings of this study to all migrants in Japan, as the characteristics of the study area were also influencing factors. In addition, the extent and nature of post-migration changes can vary with the country of origin and time of migration. Further, changes in diet and PA may have already started before migration in the country of origin due to nutrition transition. As such, post-migration changes among individuals from the same country or ethnic group could be at different levels. Therefore, we are unable to make direct and general comparisons between cultures. Furthermore, interviews were conducted by one researcher via a method the participants felt comfortable with. The fact that the interviewer is a foreign national in Japan allowed the participants to express their opinions freely without any reservations and facilitated a better interpretation of the participants’ comments by the researcher.

The study also has some limitations, mainly in relation to the generalizability of the findings, as described in the previous paragraph. The sample size of the quantitative study was small but thought to be representative of the study population as it consists of foreign residents from different parts of the world and socio-economic backgrounds. However, a small number of participants from some regions of the world limits our ability to draw conclusions based on the cultural and regional differences of immigrants. In addition, a higher proportion of the study sample was students whose dietary behaviors are generally known to be different from the dietary habits of other adults, and it might have introduced statistical bias. Further, the study used self-report measures to collect information that may have introduced recall and/or social desirability bias. However, the questionnaire was pre-tested and anonymously administered to minimize the chances of reporting bias. Another limitation of the study was the use of an online questionnaire, which may introduce response bias by favoring those who are frequent online users. However, migrants are a hard-to-reach population; thus, an online questionnaire provides a convenient way to reach them.

### 4.2. Implications for Practice and Future Research

The findings of this study shed light on important health-related behaviors among an immigrant population in Japan and can be used to inform initiatives to promote health in this rapidly growing community. For instance, health promotion and education interventions can be initiated to introduce immigrants to the local food culture and easy-to-make recipes, ways to access traditional foods and ingredients as well as health-related information, and understanding food labels and nutritional information.

Despite the limitations, the current study has the potential of expanding the research on the acculturation process and health consequences. Further, studies that aim to better understand and compare the post-migration dietary and PA changes among immigrants from different metropolitan, urban, and semi-urban regions within the country can inform stakeholders and policy makers to develop national-level support programs for newly arriving migrants.

## 5. Conclusions

In conclusion, findings from this study support the notion that changes in diet and PA have occurred among migrants in Niigata prefecture, Japan. The post-migration dietary changes are characterized by the increased intake of foods attached to Japanese cultural identity and frozen and microwavable food, and a reduced consumption of fruits. The changes in dietary behaviors and PA were mainly driven by environmental, individual, and socio-cultural factors. While our findings are in agreement with the model proposed by Satia et al. [8], qualitative data highlight the influence of study area characteristics, the duration of stay, and fluency in the Japanese language on the post-migration dietary and PA changes in the current population. The findings of this study provide a better understanding of how and why post-migration changes occur, which can help plan and implement appropriate interventions for foreign residents in Japan.

## Figures and Tables

**Table 1:** Socio-demographic characteristics of the survey and interview participants ^1^.

		Survey (*N* = 128)	Interview (*N* = 21)
Age, mean (SD)		32.6 (8.4)	33.8 (7.6)
Sex, female		74 (57.8)	14 (66.7)
Region			
	South America	5 (3.9)	2 (9.5)
	North America and Caribbean	9 (7.0)	3 (14.3)
	Africa	12 (9.4)	2 (9.5)
	South Asia	20 (15.6)	3 (14.3)
	Southeast and East Asia	59 (46.1)	6 (28.6)
	Central Asia and Russia	14 (10.9)	4 (19.0)
	Middle East and Turkey	6 (4.7)	1 (4.8)
	Australia	3 (2.3)	0
Religion			
	Christian/catholic	35 (27.3)	7 (33.3)
	Buddhist	24 (18.8)	3 (14.3)
	Islam	17 (13.3)	3 (14.3)
	Hindu	7 (5.5)	0
	No particular religious affiliation	44 (34.4)	8 (38.1)
	Other	1 (0.8)	0
Level of education			
	High school or less	7 (5.5)	0
	Vocational school/diploma	4 (3.1)	0
	Bachelor	44 (34.4)	5 (23.8)
	Master	52 (40.6)	10 (47.6)
	PhD or higher	19 (14.8)	4 (19.0)
	Other	2 (1.6)	2 (9.5)
No. of years living in Japan, mean (SD)		5.0 (6.3)	3.6 (3.0)
Residence status			
	Vocational/technical school student	3 (2.3)	0
	University student	82 (64.1)	13 (61.9)
	Employment	28 (21.9)	5 (23.8)
	Dependent	8 (6.3)	2 (9.5)
	Permanent resident	7 (5.5)	1 (4.8)
Monthly income			
	<100,000 ¥	19 (14.8)	3 (9.5)
	100,000–200,000 ¥	76 (59.4)	13 (61.9)
	200,000–300,000 ¥	20 (15.6)	3 (14.3)
	>300,000 ¥	13 (10.2)	3 (14.3)
Employment status			
	Not employed	68 (53.1)	9 (42.9)
	Part-time job/s	19 (14.8)	4 (19.0)
	Full-time job	36 (28.1)	8 (38.1)
	Self employed	5 (3.9)	0
Marital status			
	Unmarried	75 (58.6)	12 (57.1)
	Married/living together	51 (39.8)	7 (33.3)
	Divorced/widowed	2 (1.6)	2 (9.5)
Living status in Japan			
	Living alone	85 (66.4)	14 (66.7)
	Living with a partner	16 (12.5)	5 (23.8)
	Living with a family	27 (21.1)	2 (9.5)
Dietary habits ^2^			
	Vegetarian	8 (6.3)	3 (14.3)
	Vegan	4 (3.1)	1 (4.8)
	Halal	15 (11.7)	3 (14.3)
	No beef	10 (7.8)	0
	No pork	17 (13.3)	2 (9.5)
	Not any meat	1 (0.8)	0
	Nothing particular	93 (72.7)	15 (71.4)
	Other	1 (0.8)	0
Frequency of eating traditional dishes			
	Everyday	28 (21.9)	2 (9.5)
	4–6 times/week	21 (16.4)	2 (9.5)
	1–3 times/week	32 (25.0)	4 (19.0)
	Occasionally	29 (22.7)	8 (38.1)
	Rarely	18 (14.1)	5 (23.8)

^1^ Data presented as *n* (%) unless otherwise specified. ^2^ Multiple responses were allowed.

**Table 2:** Self-reported changes in food consumption since migration (*N* = 128) ^1^.

		Eat Much Less	No Significant Change	Eat Much More	Do Not Eat at All	Total Change
Staple foods						
	Rice	19 (14.8)	55 (43.0)	54 (42.2)	0	73 (57.0)
	Bread/bakery products	24 (18.8)	53 (41.1)	51 (39.8)	0	75 (58.6)
	Spaghetti/pasta	26 (20.3)	50 (39.1)	46 (35.9)	6 (4.7)	72 (56.2)
	Noodles	11 (8.6)	38 (29.7)	**74 (57.8)**	5 (3.9)	85 (66.4)
	Naan/tortilla	20 (15.6)	32 (25.0)	45 (35.2)	31 (24.2)	65 (50.8)
Legumes						
	Fresh beans/peas	31 (24.2)	44 (34.4)	34 (26.6)	19 (14.8)	65 (50.8)
	Tofu	5 (3.9)	41 (32.0)	59 (46.1)	23 (18.0)	64 (50.0)
Vegetables and fruits						
	Sweet potatoes	23 (18.0)	34 (26.6)	44 (34.4)	27 (21.1)	67 (52.3)
	Vegetables	34 (26.6)	47 (36.7)	45 (35.2)	2 (1.6)	79 (61.7)
	Green leaves	42 (32.8)	50 (39.1)	31 (24.2)	5 (3.9)	73 (57.0)
	Fruits	**64 (50.0)**	34 (26.6)	27 (21.1)	3 (2.3)	91 (71.1)
	Mushrooms	14 (10.9)	46 (35.9)	60 (46.9)	8 (6.3)	74 (57.8)
Animal foods						
	Chicken	10 (7.8)	56 (43.8)	60 (46.9)	2 (1.6)	70 (54.7)
	Beef	58 (45.3)	28 (21.9)	28 (21.9)	14 (10.9)	86 (67.2)
Seafoods						
	Raw fish	10 (7.8)	15 (11.7)	**86 (67.2)**	17 (13.3)	96 (75.0)
	Cooked/baked fish	22 (17.2)	40 (31.3)	58 (45.3)	8 (6.3)	80 (62.5)
	Seaweed	10 (7.8)	26 (20.3)	**77 (60.2)**	15 (11.7)	87 (68.0)
	Seafood	20 (15.6)	28 (21.9)	**72 (56.3)**	8 (6.3)	92 (71.9)
Dairy foods						
	Cheese	34 (26.6)	58 (45.3)	31 (24.2)	5 (3.9)	65 (50.8)
Sweets and snacks						
	Pies/cakes	46 (35.9)	47 (36.7)	32 (25.0)	3 (2.3)	78 (60.9)
	Chocolates	25 (19.5)	59 (46.1)	41 (32.0)	3 (2.3)	66 (51.5)
	Salty snacks	30 (23.4)	53 (41.4)	40 (31.3)	5 (3.9)	70 (54.7)
Beverages						
	Green tea	10 (7.8)	36 (28.1)	**75 (58.6)**	7 (5.5)	85 (66.4)
	Fruit juice	47 (36.7)	44 (34.4)	26 (20.3)	11 (8.6)	73 (57.0)
Seasoning						
	Salad dressing	11 (8.6)	51 (39.8)	63 (49.2)	3 (2.3)	74 (57.8)
	Soy sauce	13 (10.2)	38 (29.7)	**76 (59.4)**	1 (0.8)	89 (69.6)
	Miso	9 (7.0)	13 (10.2)	**100 (78.1)**	6 (4.7)	109 (85.1)
	Mayonnaise	15 (11.7)	54 (42.2)	50 (39.1)	9 (7.0)	65 (50.8)
	Sauce mix	21 (16.4)	47 (36.7)	45 (35.2)	15 (11.7)	66 (51.6)

^1^ Data presented as *n* (%). Bold numbers indicate ≥50% of change (higher or lower intake) post-migration.

**Table 3:** Self-reported changes in food preparation methods and dietary behaviors since migration (*N* = 128) ^1^.

		Less Often/Decreased	Almost Same	More Often/Increased	Not at All	Total Change
Preparation methods						
	Deep frying	35 (27.3)	45 (35.2)	30 (23.4)	18 (14.1)	65 (50.8)
	BBQ	46 (35.9)	31 (24.2)	25 (19.5)	26 (20.3)	71 (55.5)
	Grilling	43 (33.6)	33 (25.8)	29 (22.7)	23 (18.0)	72 (56.3)
	Microwaving	6 (4.7)	28 (21.9)	**85 (66.4)**	9 (7.0)	91 (71.1)
Dietary behaviors						
	Homemade meals	35 (27.3)	43 (33.6)	50 (39.1)	0	85 (66.4)
	Restaurant meals/dine-out	37 (28.9)	33 (25.8)	54 (42.2)	4 (3.1)	91 (71.1)
	Takeout	45 (35.2)	25 (19.5)	47 (36.7)	11 (8.6)	92 (71.9)
	Home delivery	53 (41.4)	16 (12.5)	13 (10.2)	46 (35.9)	67 (51.6)
	Buffet	35 (27.3)	31 (24.2)	44 (34.4)	18 (14.1)	79 (61.7)
	Skipping breakfast	16 (12.5)	30 (23.4)	53 (41.4)	29 (22.7)	69 (53.9)
	Late-night meals	17 (13.3)	24 (18.8)	58 (45.3)	29 (22.7)	75 (58.6)
	Frozen foods	18 (14.1)	32 (25.0)	**69 (53.9)**	9 (7.0)	87 (68.0)
	Organic foods	48 (37.5)	52 (40.6)	18 (14.1)	10 (7.8)	68 (51.6)

^1^ Data presented as *n* (%). Bold numbers indicate ≥50% of change (increase or decrease) post-migration.

**Table 4:** Availability, accessibility, and affordability of traditional foods/ingredients (*N* = 128).

		*n* (%)
Availability of traditional food ingredients		
	All are commonly available	8 (6.3)
	Many are commonly available	56 (43.8)
	Limited options are available	63 (49.2)
	Not available at all	1 (0.8)
Affordability of traditional food ingredients		
	Expensive, do not usually buy	47 (36.7)
	Regardless of high prices, I usually buy	51 (39.8)
	Affordable	28 (21.9)
	Cheap	2 (1.6)
Methods of buying traditional food ingredients ^1^		
	Local shops located in the neighborhood	42 (32.8)
	International shops located within Niigata prefecture	73 (57.0)
	Online orders within Japan	76 (59.4)
	Online orders (international)	25 (19.5)
	Sent by family members from the home country	43 (33.6)
	Do not buy at all	12 (9.4)
Availability of traditional restaurants		
	There are many, I usually go	4 (3.1)
	There are some, I usually go	13 (10.2)
	There are some, I occasionally go	39 (30.5)
	There are none	72 (56.3)

^1^ Multiple responses were allowed.

**Table 5:** Self-reported changes in physical activities (*N* = 128) ^1^.

	Less Often/Decreased	Almost Same	More Often/Increased	Not at All	Total Change
Household chores	10 (7.8)	43 (33.6)	71 (55.5)	4 (3.1)	**81 (63.3)**
Gardening	31 (24.2)	20 (15.6)	12 (9.4)	65 (50.8)	43 (33.6)
Walking	17 (13.3)	24 (18.8)	83 (64.8)	4 (3.1)	**100 (78.1)**
Climbing stairs	16 (12.5)	39 (30.5)	64 (50.0)	9 (7.0)	**80 (62.5)**
Jogging	19 (14.8)	41 (32.0)	25 (19.5)	43 (33.6)	44 (34.3)
Cycling	23 (18.0)	15 (11.7)	59 (46.1)	31 (24.2)	**82 (64.1)**
Running	25 (19.5)	27 (21.1)	21 (16.4)	55 (43.0)	46 (35.9)
Workout at home	21 (16.4)	41 (32.0)	25 (19.5)	41 (32.0)	46 (35.9)
Workout at gym	17 (13.3)	21 (16.4)	18 (14.1)	72 (56.3)	35 (27.4)
Yoga/Pilates	12 (9.4)	19 (14.8)	8 (6.3)	89 (69.5)	20 (15.7)
Swimming	26 (20.3)	16 (12.5)	7 (5.5)	79 (61.7)	33 (25.8)

^1^ Data presented as *n* (%). Bold numbers indicate ≥50% of total change.

**Table 6:** Post-migration changes and adaptive behaviors related to the diet and PA.

How?	Why?
Changes/adaptive behaviors related to food purchasing	
1. Opt for cheaper foods 2. Purchase only when the prices are lower	- Higher/lower prices of foods and ingredients
3. Buying smaller quantities	- Higher/lower prices of foods and ingredients - Time restrictions and busy lifestyle - Living alone and changed family structure
4. Driving to other cities where international stores are available 5. Ordering online	- Availability and accessibility of foods and ingredients
6. Buying readily available meals from convenience stores	- Perceived food safety - Availability and accessibility - Time restrictions and busy lifestyle
7. Avoid purchasing some products	- Lack of or difficulty in understanding the product information (ex: ingredients) - Difference in flavor or quality compared to the traditional food
8. Start to procure foods and ingredients	- Living alone and changed family structure
9. Choosing foods with similar tastes or qualities to traditional foods	- Preference of traditional foods/tastes
10. Checking the ingredients of foods before purchasing	- Religious influence - Ingrained habits and beliefs - Food safety considerations
Changes/adaptive behaviors related to food preparation	
1. Start to prepare own meals	- Cost of eating outside - Living alone and changed family structure - Difference between pre- and post-migration lifestyles
2. Prepare simple and non-complicated meals	- The burden of preparing complicated or time-consuming meals for one person (living alone) - Time restrictions and busy lifestyle
3. Fusion of Japanese and traditional cuisines	- Family structure (foreign–Japanese households) - Availability and accessibility of foods and ingredients - Time restrictions and busy lifestyle - Food preferences and limitations - The burden of preparing complicated traditional meals for one person (living alone)
4. Use of substitute ingredients for unavailable traditional ingredients 5. Ignore the non-availability of ingredients that do not affect the flavor significantly 6. Giving up preparation of some traditional dishes 7. Acquiring the skills to prepare and cook traditional/international dishes independently	- Availability and accessibility of foods and ingredients
8. Not preparing foods that are unavailable in the home country	- Lack of knowledge/information on preparation methods
9. Bulk food preparation and storing 10. Choosing foods that do not spoil quickly	- Time restrictions and busy lifestyle
Changes/adaptive behaviors related to food intake/dietary habits	
1. Bi-cultural meal patterns (traditional cuisines of the foreign resident, and Japanese cuisines)	- Family structure (foreign–Japanese households) - Food preferences and limitations
2. Increased intake of Japanese food 3. Learn about authentic Japanese food culture	- Newfound preference for Japanese foods/tastes - Health-related factors - Affordability, availability, and accessibility of foods and ingredients - Influence of the Japanese food culture - Relationships with Japanese people - Willingness to try new foods
4. Limited intake of Japanese foods	- Taste preferences - Religious influence - Health-related factors - Ingrained beliefs
5. Not following meal-associated practices that were enjoyed with the family at the home country (ex: tea time)	- Changed family structure - Time restrictions and busy lifestyle
6. Increased food intake	- Climate in Niigata
7. Missing meals (breakfast)	- Time restrictions and busy lifestyle
8. Frequent meals	- Features of Japanese food culture (light meals) - Climate in Niigata
9. Eating healthier/unhealthier than in the home country	- Contrast between traditional and Japanese food cultures - Ingrained habits and beliefs
10. Increased use of frozen foods	- Time restrictions and busy lifestyle - Availability and accessibility
11. Limiting or reduced opportunities to eat together/eat out	- Climate in Niigata - Changes in family structure/living status - Religious influence - Effect of the COVID-19 pandemic
12. Pre-migration changes in the diet in preparation for adapting to Japanese (food) culture	- Notions regarding Japanese (food) culture
Changes/adaptive behaviors related to PA	
1. Reduced outdoor PA (going out and cardio workouts)	- Climate in Niigata - Changes in social life
2. Opting for indoor PA (weight lifting and push-ups)	- Climate in Niigata
3. Increased outdoor PA	- Health-conscious lifestyle in Japan - Perceived safety
4. Sedentary lifestyle	- Changes in social life - Work environment in Japan - Small apartments (limited movements inside the home)

## Data Availability

The data used and/or analyzed during the current study are available from the corresponding author upon reasonable request. The qualitative data are not publicly available due to ethical considerations.

## Supplementary Materials

The following supporting information can be downloaded at: https://www.mdpi.com/article/10.3390/nu15163639/s1, Supplementary File S1: Table S1.1: Participant representation from each country (p. 1); Table S1.2: Associations between dietary habits and frequency of traditional food consumption (p. 2); Table S1.3: Self-reported changes in consumption of foods/food groups (pp. 2–3); Table S1.4: Associations between post-migration fruit consumption and monthly income (p. 3); Table S1.5: Self-reported changes in food preparation methods and dietary behaviors (p. 4); Table S1.6: Increased use of frozen foods and microwaving by region and residence status (pp. 4–5); Table S.1.7: Associations between the availability of traditional food ingredients and frequency of traditional food consumption (p. 5); Table S1.8: Post-migration changes in Noodle consumption by region (p. 5); Table S1.9: Post-migration changes in Green tea consumption by region (p. 5). Supplementary File S2: Table S2.1: Factors influencing post-migration diet changes (pp. 1–8); Table S2.2: Factors influencing changes in PA since migration (pp. 8–9). Supplementary File S3: Figure S1: Thematic Map: Environmental factors influencing post-migration dietary changes (p. 1); Figure S2: Thematic Map: Individual factors influencing post-migration dietary changes (p. 2); Figure S3: Thematic Map: Socio-cultural factors influencing post-migration dietary changes (p. 3); Figure S4: Thematic Map: Factors influencing post-migration PA changes (p. 4).
