# Supplementary material for: Post-Migration Changes in Dietary Patterns and Physical Activity among Adult Foreign Residents in Niigata Prefecture, Japan: A Mixed-Methods Study
Source: Nutrients. 2023 Aug 19;15(16):3639. doi: 10.3390/nu15163639 (PMC10458835; doi:10.3390/nu15163639)
Supplement: Supplementary file 1 [file nutrients-15-03639-s001.zip › Supplementary File S1.pdf]

Table S1.1: Participant representation from each country[1]

	Survey (N=128)	Interview (N=21)
South America		
Brazil	1 (0.8)	0
Chile	1 (0.8)	1 (5.0)
Colombia	1 (0.8)	0
Peru	2 (1.6)	1 (5.0)
North America and Caribbean		
USA	6 (4.7)	2 (10.0)
Mexico	2 (1.6)	1 (5.0)
Jamaica	1 (0.8)	0
Africa		
Benin	1 (0.8)	0
Côte d'Ivoire	1 (0.8)	1 (5.0)
Malawi	2 (1.6)	0
Mauritius	1 (0.8)	1 (5.0)
Mozambique	3 (2.3)	0
Namibia	1 (0.8)	0
Nigeria	1 (0.8)	0
Rwanda	1 (0.8)	0
Tanzania	1 (0.8)	0
South Asia		
Bangladesh	2 (1.6)	0
India	3 (2.3)	1 (5.0)
Nepal	4 (3.1)	0
Sri Lanka	11 (8.6)	2 (10.0)
Southeast and East Asia		
Indonesia	10 (7.8)	2 (5.0)
Laos	1 (0.8)	0
Malaysia	3 (2.3)	0
Myanmar	6 (4.7)	1 (5.0)
Philippines	8 (6.3)	1 (5.0)
Thailand	6 (4.7)	0
Vietnam	5 (3.9)	1 (5.0)
China	9 (7.0)	1 (5.0)
South Korea	3 (2.3)	0
Mongolia	3 (2.3)	0
Taiwan	5 (3.9)	0
Central Asia and Russia		
Kazakhstan	2 (1.6)	2 (10.0)
Russia	12 (9.4)	2 (10.0)
Middle East and Turkey		
Egypt	1 (0.8)	0
Jordan	1 (0.8)	0
Turkey	4 (3.1)	1 (5.0)
Australia	3 (2.3)	0

[1] Data presented as *n* (%).

Table S1.2: Associations between dietary habits and frequency of traditional food consumption[1]

	More than 4 times/ week (*N*=49)	Less than 4 times/ week (*N*=79)
Vegan	1 (2.0)	3 (3.8)
Vegetarian	3 (6.1)	5 (6.3)
Halal	6 (12.2)	9 (11.4)
No beef	8 (16.3)	2 (2.5)[a]
No pork	9 (18.4)	8 (10.1)
No any meat	1 (2.0)	0
Nothing particular	30 (61.2)	63 (79.7)[b]

[1]Data presented as *n* (% within the frequency); [a] χ^2 (1, *N*=128) = 7.991, $p = 0.007$; [a] χ^2 (1, *N*=128) = 5.222, $p = 0.022$

Table S1.3: Self-reported changes in consumption of foods/ food groups (*N*=128)[1]

	**Eat much less**	**No significant change**	**Eat much more**	**Don't eat at all**	**Total change**
Staple foods					
Rice	19 (14.8)	55 (43.0)	54 (42.2)	0	**73 (57.0)**
Bread and bakery products	24 (18.8)	53 (41.4)	51 (39.8)	0	**75 (58.6)**
Ready-to-eat-cereals	35 (27.3)	27 (21.1)	26 (20.3)	40 (31.3)	62 (47.6)
Spaghetti/ pasta	26 (20.3)	50 (39.1)	46 (35.9)	6 (4.7)	**72 (57.2)**
Noodle	11 (8.6)	38 (29.7)	**74 (57.8)**	5 (3.9)	**85 (66.4)**
Naan/ tortilla	20 (15.6)	32 (25.0)	45 (35.2)	31 (24.2)	**65 (50.8)**
Pizza	30 (23.4)	53 (41.4)	32 (25.0)	13 (10.2)	62 (48.4)
Pancake/ waffles	34 (26.6)	40 (31.3)	20 (15.6)	34 (26.6)	54 (42.2)
Legumes					
Fresh beans/ peas	31 (24.2)	44 (34.4)	34 (26.6)	19 (14.8)	**65 (50.8)**
Dried beans/ peas	34 (26.6)	48 (37.5)	17 (13.3)	29 (22.7)	51 (40.0)
Tofu	5 (3.9)	41 (32.0)	59 (46.1)	23 (18.0)	**64 (50.0)**
Vegetables and fruits					
Potatoes	30 (23.4)	62 (48.4)	32 (25.0)	4 (3.1)	62 (48.4)
Sweet potatoes	23 (18.0)	34 (26.6)	44 (34.4)	27 (21.1)	**67 (52.3)**
Yams	28 (21.9)	36 (28.1)	18 (14.1)	46 (35.9)	47 (36.0)
Vegetables	34 (26.6)	47 (36.7)	45 (35.2)	2 (1.6)	**79 (61.7)**
Green leaves	42 (32.8)	50 (39.1)	31 (24.2)	5 (3.9)	**73 (57.0)**
Pickled vegetables	24 (18.8)	41 (32.0)	39 (30.5)	24 (18.8)	63 (49.2)
Fruits	**64 (50.0)**	34 (26.6)	27 (21.1)	3 (2.3)	**91 (71.1)**
Mushrooms	14 (10.9)	46 (35.9)	60 (46.9)	8 (6.3)	**74 (57.8)**
Animal foods					
Chicken	10 (7.8)	56 (43.8)	60 (46.9)	2 (1.6)	**70 (54.7)**
Pork	22 (17.2)	40 (31.3)	41 (32.0)	25 (19.5)	63 (49.2)
Beef	58 (45.3)	28 (21.9)	28 (21.9)	14 (10.9)	**86 (67.2)**
Sausage	32 (25.0)	45 (35.2)	24 (18.8)	27 (21.1)	56 (43.8)
Ham	28 (21.9)	39 (30.5)	21 (16.4)	40 (31.3)	49 (38.3)
Bacon	25 (19.5)	42 (32.8)	18 (14.1)	43 (33.6)	43 (33.6)
Eggs	6 (4.7)	67 (52.3)	54 (42.2)	1 (0.8)	60 (46.9)
Seafoods					

Raw fish	10 (7.8)	15 (11.7)	**86 (67.2)**	17 (13.3)	**96 (75.0)**
Cooked/ baked fish	22 (17.2)	40 (31.3)	58 (45.3)	8 (6.3)	**80 (62.5)**
Sea weed	10 (7.8)	26 (20.3)	**77 (60.2)**	15 (11.7)	**87 (68.0)**
Sea food	20 (15.6)	28 (21.9)	**72 (56.3)**	8 (6.3)	**92 (71.9)**
Dairy foods					
Milk	24 (18.8)	68 (53.1)	26 (20.3)	10 (7.8)	50 (39.1)
Yoghurt	33 (25.8)	59 (46.1)	30 (23.4)	6 (4.7)	63 (49.2)
Cheese	34 (26.6)	58 (45.3)	31 (24.2)	5 (3.9)	**65 (50.8)**
Butter	37 (28.9)	69 (53.9)	15 (11.7)	7 (5.5)	52 (40.6)
Sweets and snacks					
Pies/ Cakes	46 (35.9)	47 (36.7)	32 (25.0)	3 (2.3)	**78 (60.9)**
Chocolates	25 (19.5)	59 (46.1)	41 (32.0)	3 (2.3)	**66 (51.5)**
Candy	35 (27.3)	65 (50.8)	12 (9.4)	16 (12.5)	47 (36.7)
Ice-cream	22 (17.2)	63 (49.2)	36 (28.1)	7 (5.5)	58 (45.3)
Salty snacks	30 (23.4)	53 (41.4)	40 (31.3)	5 (3.9)	**70 (54.7)**
Beverages					
Coffee	8 (6.3)	51 (39.8)	50 (39.1)	19 (14.8)	58 (45.3)
Green tea	10 (7.8)	36 (28.1)	**75 (58.6)**	7 (5.5)	**85 (66.4)**
Other tea types	20 (15.6)	60 (46.9)	35 (27.3)	13 (10.2)	55 (42.9)
Fruit Juice	47 (36.7)	44 (34.4)	26 (20.3)	11 (8.6)	**73 (57.0)**
Soft drinks	21 (16.4)	63 (49.2)	31 (24.2)	13 (10.2)	52 (40.6)
Soup/ porridge	35 (27.3)	59 (46.1)	27 (21.1)	7 (5.5)	62 (48.4)
Sport/ energy drink	6 (4.7)	41 (32.0)	27 (21.1)	54 (42.2)	33 (25.8)
Alcohol	11 (8.6)	45 (35.2)	33 (25.8)	39 (30.5)	44 (34.4)
Seasoning					
Salad dressing	11 (8.6)	51 (39.8)	63 (49.2)	3 (2.3)	**74 (57.8)**
Soy sauce	13 (10.2)	38 (29.7)	**76 (59.4)**	1 (0.8)	**89 (69.6)**
Miso	9 (7.0)	13 (10.2)	**100 (78.1)**	6 (4.7)	**109 (85.1)**
Ketch-up	23 (18.0)	57 (44.5)	30 (23.4)	18 (14.1)	53 (41.4)
Mayonnaise	15 (11.7)	54 (42.2)	50 (39.1)	9 (7.0)	**65 (50.8)**
Sauce mix	21 (16.4)	47 (36.7)	45 (35.2)	15 (11.7)	**66 (51.6)**

[1]Data presented as *n* (%).
Bold numbers indicate ≥50% of total change.

Table S1.4: Associations between post-migration fruit consumption and monthly income[1]

**Monthly income** / **Fruit intake**	<100,000¥	100,000 – 200,000¥	200,000 – 300,000¥	>300,000¥	
Eat much less	12 (63.2)	41 (53.9)	6 (30.0)	5 (38.5)	χ^2 (1, *N*=128) = 25.467, *p* = 0.002
No significant change	3 (15.8)	14 (18.4)	10 (50.0)	7 (53.8)	
Eat much more	2 (10.5)	21 (27.6)	3 (15.0)	1 (7.7)	
Don't eat at all	2 (10.5)	0	1 (5.0)	0	

[1] Data presented as *n* (% within monthly income).

Table S1.5: Self-reported changes in food preparation methods and dietary behaviors (N=128)[1]

	**Less often/ Decreased**	**Almost same**	**More often/ Increased**	**Not at all**	**Total change**
Preparation methods					
Stir-frying	12 (9.4)	70 (54.7)	40 (31.3)	6 (4.7)	52 (40.7)
Deep frying	35 (27.3)	45 (35.2)	30 (23.4)	18 (14.1)	**65 (50.8)**
BBQ	46 (35.9)	31 (24.2)	25 (19.5)	26 (20.3)	**71 (55.5)**
Grilling	43 (33.6)	33 (25.8)	29 (22.7)	23 (18.0)	**72 (56.3)**
Baking	38 (29.7)	32 (25.0)	13 (10.2)	45 (35.2)	51 (39.8)
Microwaving	6 (4.7)	28 (21.9)	**85 (66.4)**	9 (7.0)	**91 (71.1)**
Stewing	17 (13.3)	66 (51.6)	31 (24.2)	14 (10.9)	48 (37.5)
Dietary behaviors					
Homemade meals	35 (27.3)	43 (33.6)	50 (39.1)	0	**85 (66.4)**
Restaurant meals/ dine-out	37 (28.9)	33 (25.8)	54 (42.2)	4 (3.1)	**91 (71.1)**
Takeout	45 (35.2)	25 (19.5)	47 (36.7)	11 (8.6)	**92 (71.9)**
Home delivery	53 (41.4)	16 (12.5)	13 (10.2)	46 (35.9)	**67 (51.6)**
Buffet	35 (27.3)	31 (24.2)	44 (34.4)	18 (14.1)	**79 (61.7)**
Skipping breakfast	16 (12.5)	30 (23.4)	53 (41.4)	29 (22.7)	**69 (53.9)**
Skipping lunch/ dinner	15 (11.7)	44 (34.4)	33 (25.8)	36 (28.1)	48 (37.5)
Late night meals	17 (13.3)	24 (18.8)	58 (45.3)	29 (22.7)	**75 (58.6)**
Meal frequency	23 (18.0)	80 (62.5)	23 (18.0)	2 (1.6)	46 (36.0)
Fasting/ Intermittent fasting	19 (14.8)	39 (30.5)	23 (18.0)	47 (36.7)	42 (32.8)
Snacking between meals	21 (16.4)	61 (47.7)	36 (28.1)	10 (7.8)	57 (44.5)
Canned foods	30 (23.4)	48 (37.5)	32 (25.0)	18 (14.1)	62 (48.4)
Frozen foods	18 (14.1)	32 (25.0)	**69 (53.9)**	9 (7.0)	**87 (68.0)**
Organic foods	48 (37.5)	52 (40.6)	18 (14.1)	10 (7.8)	**68 (51.6)**

[1] Data presented as n (%).
Bold numbers indicate ≥50% of total change.

Table S1.6: Increased use of frozen foods and microwaving by region and residence status[1]

	**Frozen foods (N=69)**	**Microwaving (N=85)**
Region		
South America	2 (40.0)	4 (80.0)
North America and Caribbean	2 (22.2)	4 (44.4)
Africa	7 (58.3)	8 (66.7)
South Asia	13 (65.0)	17 (85.0)
Southeast Asia	28 (71.8)	32 (82.1)
East Asia	8 (40.0)	9 (45.0)
Central Asia and Russia	3 (21.4)	4 (28.6)
Middle East and Turkey	6 (100.0)	6 (100.0)
Australia	0	1 (33.3)
Residence status		

Vocational/ technical school student	2 (66.7)	3 (100.0)
University student	53 (64.6)	62 (75.6)
Employment	8 (28.6)	10 (35.7)
Dependent	2 (25.0)	5 (62.5)
Permanent resident	4 (57.1)	5 (71.4)

[1]Data presented as *n* (% within region or residence status)

Table S1.7: Associations between the availability of traditional food ingredients and frequency of traditional food consumption[1]

**Availability** / **Frequency**	Available	No/ limited availability	
More than 4 times/ week	30 (61.2)	19 (38.8)	χ^2 (1, N=128) = 4.001, p = 0.045
Less than 4 times/ week	34 (43.0)	45 (57.0)	

[1] Data presented as *n* (% within frequency).

Table S1.8: Post-migration changes in Noodle consumption by region[1]

	**Eat much less (*N*=11)**	**No significant change (*N*=38)**	**Eat much more (*N*=74)**	**Eat much less (*N*=5)**
South America	0	0	**5 (100.0)**	0
North America and Caribbean	0	0	**9 (100.0)**	0
Africa	2 (16.7)	1 (8.3)	8 (66.7)	1 (8.3)
South Asia	4 (20.0)	1 (10.0)	14 (70.0)	0
Southeast Asia	3 (7.7)	13 (33.3)	20 (51.3)	3 (7.7)
East Asia	1(5.0)	14 (70.0)	5 (25.0)	0
Central Asia and Russia	1 (7.1)	4 (28.6)	9 (64.3)	0
Middle East and Turkey	0	4 (66.7)	2 (33.3)	0
Australia	0	0	2 (66.7)	1 (33.3)

[1]Data presented as *n* (%).
Bold numbers indicate ≥50% of increased consumption within the region.

Table S1.9: Post-migration changes in Green tea consumption by region[1]

	**Eat much less (*N*=10)**	**No significant change (*N*=36)**	**Eat much more (*N*=75)**	**Eat much less (*N*=7)**
South America	0	3 (60.0)	2 (40.0)	0
North America and Caribbean	0	2 (22.2)	4 (44.4)	3 (33.3)
Africa	2 (16.7)	5 (41.7)	5 (41.7)	0
South Asia	3 (15.0)	1 (5.0)	**15 (75.0)**	1 (5.0)
Southeast Asia	1 (2.6)	8 (20.5)	**28 (71.8)**	2 (5.1)
East Asia	1(5.0)	9 (45.0)	10 (50.0)	0
Central Asia and Russia	1 (7.1)	6 (42.9)	7 (50.0)	0
Middle East and Turkey	2 (33.3)	1 (16.7)	2 (33.3)	1 (16.7)
Australia	0	1 (33.3)	2 (66.7)	0

[1]Data presented as *n* (%).
Bold numbers indicate ≥50% of increased consumption within the region.
