# Supplementary material for: Post-Migration Changes in Dietary Patterns and Physical Activity among Adult Foreign Residents in Niigata Prefecture, Japan: A Mixed-Methods Study
Source: Nutrients. 2023 Aug 19;15(16):3639. doi: 10.3390/nu15163639 (PMC10458835; doi:10.3390/nu15163639)
Supplement: Supplementary file 1 [file nutrients-15-03639-s001.zip › Supplementary File S2.pdf]

Table S2.1: Factors influencing post-migration diet changes

Categories	Factors	Subthemes (bold) and illustrative Quotes (italics)
Environmental factors	Affordability of foods and ingredients	**1. Higher or lower prices of foods/ meals**<br>***Higher prices of certain foods, mainly, fruits, vegetables, meat (beef), and traditional ingredients, compared to home countries.**<br>***Lower prices of local foods such as *natto* (fermented soybeans), tofu**<br>*"Actually, in Chile, vegetables and fruits are way cheaper than here. Here [in Japan], super expensive for me…For example, in my daily life [in Chile], I used to eat a lot of peaches and avocados. But, here in Japan, it decreased because it is too expensive."* (Interview #1, Female, Chile)<br>*"I consume [in Japan] a lot of chicken and some pork…..and beef is only when it is half price, I used to eat a lot of beef in Mexico. I used to prepare big steaks for myself. But here, I cannot. I cannot afford it. Also, I eat a lot of Natto (Fermented soybeans), like maybe 2 or 3 packs per day on a regular basis. It is super cheap, so I can buy a lot and no problem."* (Interview #18, Male, Mexico)<br><br>**2. Adaptive purchasing and dietary behaviors**<br>**The practice of choosing foods that are cheaper, buying in smaller quantities or when the prices are lower, and preparing own meals rather than eating outside.**<br>*"Fruit intake has reduced a little bit and I'm compensating with more vegetables because vegetables are more affordable [than fruits] although the choices are less."* (Interview #8, Female, Mauritius)<br>*"I don't think I eat more meat here because it is quite expensive and quite hard to find halal meat. So, I try to eat more vegetables than chicken or meat here. Here the prices of spices are quite expensive for me, so I don't use that much spices."* (Interview #17, Female, Indonesia)<br>*"In my country, I am not so familiar with cooking. But, in Japan, it's mandatory [cooking] if I like to make a saving. Because eating outside many times is a little bit expensive."* (Interview #7, Male, Côte d'Ivoire)
	Availability and accessibility of foods and ingredients	**1. Limited/ wide availability of foods or ingredients**<br>***Some foods or ingredients are not/ less available (vegetarian foods, traditional spices, halal food). Limited availability of foreign food restaurants.**<br>*"Here, <u>vegetarian options</u> are too less. I always feel like the restaurants should become more vegetarian friendly. I think every restaurant should have at least one option."* (Interview #2, Male, India)<br>*"In Myanmar, <u>vegetables like roselle, tamarind, and the water spinach</u>, are available year around. But, those kind of vegetables are not grown in Japan, as I think."* (Interview #9, Female, Myanmar)<br>*"In California there are all kind of fast-<u>food restaurants</u> and here its limited, like here there are only handful of*

		Mexican restaurants but there's whole bunch in America. There are few Indian curry places here and not lots of Thai food, and I have to eat lots of Japanese foods because that what available." (Interview #11, Male, USA)<br>*"It is hard to find Indonesian spices in local markets; for example, I cannot find lemongrass or spicy chilies."* (Interview #17, Female, Indonesia)<br><br>*** Some foods or ingredients are difficult to buy in the same quantities or qualities (beef, traditional flavors)**<br>*"Some foods, for example, beef, are less available. Because, here [in Japan] beef is like very thin cuts. Back home, we buy the whole piece, like 5kg of beef."* (Interview #1, Female, Chile)<br>*"When we go to Sri Lankan restaurants [in Japan], the real Sri Lankan taste is not there, the flavor is different from what we know. I think it's because the curries have been modified to the Japanese taste. Even if we ask for the spiciest foods, it's not spicy."* (Interview #15, Male, Sri Lanka)<br><br>***Challenges in accessing traditional foods and ingredients**<br>*"In Tokyo, there is a restaurant each Sunday to cook our country food. But I didn't go there even one time. Because it's far from me. But it's [traditional restaurant] not available here in Niigata."* (Interview #7, Male, Côte d'Ivoire)<br>"It's *quite hard to find the spices, and I know only one shop which has Indonesian spices and halal meats. But it is hard for me to get to the shop every day because it is quite far."* (Interview #17, Female, Indonesia)<br><br>***Wide availability of some foods and ingredients (tofu, mushroom)**<br>*"Tofu, we don't eat like here in Japan. I was surprised of the variety of tofu they have here and it is very cheap also. Back home it is more expensive."* (Interview #1, Female, Chile)<br>*"In Japan, mushroom is common, we can see a variety of mushrooms in Japan. But they are expensive in Myanmar. Also, there is no frozen foods in Myanmar. But in here, I mostly use the frozen foods."* (Interview #9, Female, Myanmar)<br>*"Availability is a very big factor for my diet changes. I like chicken inners, like heart, or stomach, which are easily available in Japan. In Russia or Canada, I have to find them, like look for stores and find them, but in Japan, when you go to a supermarket, it's there, and it's cheap. Also, I haven't seen daikon (Japanese radish) before coming here, but now daikon is a huge part of my diet. You can do so many things with it."* (Interview #10, Female, Russia)<br><br>***Convenient access to certain foods (convenience stores, bake shops)**<br>*"Here [in Japan] things are available in convenience stores all the time. Even if you don't cook, you can just go and grab something."* (Interview #2, Male, India)<br>*"In Japan, there are a lot of bake shops readily available for people who are hungry or want a quick bite, and the price is cheaper than buying a meal."* (Interview #20, Female, Philippines)

		**2. Adaptive purchasing and dietary behaviors**<br>***Use of substitute ingredients for unavailable traditional ingredients**<br>*"If I cannot find Myanmar spices, I use Korean spice powder. Yes, but it is just a substitute."* (Interview #9, Female, Myanmar)<br><br>*** Fusion of Japanese and traditional cuisines**<br>*"Since we can't get all the stuff that we would have in America, my wife would add Japanese stuff like a fusion."* (Interview #11, Male, USA)<br><br>***Ignoring the non-availability of ingredients that do not affect the flavor significantly**<br>*"When we make dhal curry in Sri Lanka, we always use fresh curry leaves and pandan. In Japan, we can't find those or use any substitutes for them. But the unavailability of those doesn't make any huge taste different, so we can ignore it and cook dhal in our own way."* (Interview #15, Male, Sri Lanka)<br><br>***Giving up preparation of some traditional dishes**<br>*"If there is big effect in flavor wise when something is not available, then we are not preparing those foods."* (Interview #16, Male, Sri Lanka)<br>*"I try to substitute first, but if I cannot find the substitutes, I will just give up."* (Interview #17, Female, Indonesia)<br><br>***Driving to other cities where international stores are available**<br>*"There aren't a lot of, especially stalls or facilities that really cater to the international community [in Niigata]. We go to COSTCO in Yamagata (a nearby prefecture) quite often, at least once every 5 or 6 weeks."* (Interview #5, Male, USA)<br><br>***Ordering online**<br>*"Traditional ingredients, we always order from Tokyo. Prices are high, but if I want to eat Turkish food, you say, "ahh, just for one time, so it's okay."* (Interview #14, Female, Türkiye)<br><br>***Acquiring the skills to prepare traditional/ international dishes independently**<br>*"There's only one real Mexican restaurant here and it was mostly closed during the pandemic. Because of this, I made my own Mexican food at home. Also, there has never been a store or restaurant where you can buy a quality meat pie. So, I learned how to make those as well. Also, I like Doner kebabs, and even though there are some Turkish restaurants, they don't serve the traditional lamb/ beef meat mix. This is possibly because it is quite expensive or because of local tastes. So, I also learned how to process that meat myself."* (Interview #5, Male, USA)
	Food safety and	**1. Perceived safety/ unsafety about foods and ingredients in Japan compared to the home country**

	related information	*"One good thing is that after coming to Japan, I never felt any stomach upset or food poisoning. They [in Japan] don't keep any food which is out of date. They just throw it as soon as possible."* (Interview #2, Male, India)<br>*"I think in Japan, the quality and cleanliness of food is better. In convenience store food, there is a time of expiration and they remove those foods before that time. In Sri Lanka, they always cover the food and we cannot actually see the food. But, here in Japan, you can see what is inside the bento (Japanese lunch box)."* (Interview #15, Male, Sri Lanka)<br><br>**2. Lack of food-related information or knowledge**<br>*"It is very hard to understand the ingredients of many products in the supermarket and the list is not detailed enough and quite vague eg. Preservatives, flavor enhancers, etc. So, there is a certain amount of fear when buying products but since we can't do much about it, we kind of give up."* (Survey open comments, Female, Australia)<br>*"We don't have much knowledge on how to prepare green leaf varieties available here [in Japan]."* (Interview #16, Male, Sri Lanka)
	Climate	**Cold weather in Niigata**<br>*"Because of the weather and also the Japanese culture, incorporating tea with every meal has been another change that happened."* (Interview #20, Female, Philippines)
Individual factors	Family structure/ living status	**1. Burden of preparing complicated or time-consuming meals for one person**<br>*"For my 6 years in Japan, only once I cooked borscht. I didn't want to deal with that kind of problem, because, it's only me. I ask myself, 'can I survive without it?', then, no problem."* (Interview #4, Female, Russia)<br>*"Actually, when I was in Turkey, I wasn't cooking anything……….. Here, I am just one person, so I don't want to cook a whole meal, like soup and all [as back in Turkey], It doesn't really matter for me."* (Interview #14, Female, Türkiye)<br><br>**2. Bi-cultural diet pattern in Japanese-foreign households**<br>*"In our house, my wife is Japanese, and we often have Japanese night, where we will eat Japanese food. Another night she'll say, 'Do you want to cook tonight' I'll say, 'Yeah,' and we will have sausage rolls or maybe Buffalo wings or something else I enjoy making. So, the pattern is that we vary our diet on different days between Japanese food and international food that we like and I have learned to make over the years."* (Interview #5, Male, USA·)<br><br>**3. Newfound responsibility of procuring and preparing foods**<br>*"In my country, I cooked only one meal per day and my mother cooks the other meal as we shared the responsibility. Also in my country, if I don't want to cook, I can order food or I can eat in the canteen at work but here, every day I have prepared my own food."* (Interview #8, Female, Mauritius)<br>*"I started to cook after coming here, before that I didn't cook…just eat what my mother cooked or buy from shops, because it is cheap there."* (Interview #21, Female, China)

		**4. Changes in meal-associated family traditions**<br>*"In Chile, I used to have breakfast, lunch, then tea time, which is around 6 PM, and then dinner, which is around 9 or 10 PM. But here in Japan, I changed to 3, to breakfast, lunch, and dinner. Tea time [as in Chile], I don't do it anymore. Actually, back home, it was fun. Because we do it as a family, and we all sit together and drink hot tea with bread or some cake."* (Interview #1, Female, Chile)
	Food preferences and limitations	**1. Preference for the traditional foods**<br>***Missing traditional foods/ tastes**<br>*"In Japan, I'm not a big fan of sweets and I don't think that sweets are good as in America, may be America uses more sugar. I also don't like Japanese pizza that much because they usually get it thin and I like thick pizza. So, I miss different pizzas and hamburgers and I don't think it's good as America."* (Interview #11, Male, USA)<br><br>***Avoiding Japanese products with flavors different from traditional products**<br>*"The taste of the yogurt [in Japan] is not like the one I used to eat. I guess ours [in Turkey] is more salty and more creamy. Yeah, like one time I bought yogurt, and I didn't realize It wasn't a plain one, so it's just sugary. So, I was so disappointed. I was like, why I didn't read the label? So, my usage of milk products is decreasing, cheese also, yeah."* (Interview #14, Female, Türkiye)<br><br>*** Choosing foods with similar tastes or qualities to traditional foods**<br>*"I used to eat meat every day. I don't eat meat every day [in Japan], because it is expensive here. Also, the flavor is not like Kazakhstan. Kazakhstan meat is really good meat, I feel it is fresh; you kind of know that it is better. That's why I prefer to eat fish because it is fresh here."* (Interview #19, Female, Kazakhstan)<br><br>**2. Newfound preference for Japanese foods/ tastes**<br>*"I eat more rice [in Japan]. I think the rice in Japan is very delicious."* (Interview #13, Female, Kazakhstan)<br>*"I am preparing my own miso now because I find it healthy and tasty and I'm trying to eat Japanese food but in my way of cooking."* (Interview #8, Female, Mauritius)<br><br>**3. Willingness to try new foods**<br>*"I am not so strict about food. I can eat any food, so even if I am in a foreign place, I can become familiar to the food from that place. So, it is not mandatory for me to look for my own country foods here [in Japan]. But, if I can get it, I can eat. But if not, it's not a problem for me. I am not so sure that there are foods I don't like."* (Interview #7, Male, Côte d'Ivoire)

		**4. Ingrained habits and beliefs**<br>*"I would say that, in general, for me…..I am like conservative. Therefore, either I am here or I am in Russia, my habits quite similar. For example, if I drink 2 cups of coffee here, I drink the same amount in Russia. It's not that different. Of course, there are some differences due to culture and country. But again, if I want some fruits, I eat fruits either here or over there."* (Participant #4, Female, Russia)<br>*"Here [in Japan], I prefer to cook by myself because I don't know what is going on with the foods that I buy. I think it is too chemical or artificial. So, I prefer a nice restaurant or home."* (Interview #19, Female, Kazakhstan)<br><br>**5. Health-related factors**<br>*"Rice [in Japan] is very different, I mean.. we don't have sticky rice in Sri Lanka, we either eat red rice or white rice. When I started to eat Japanese rice, I got gastritis. So, now I eat less Japanese rice and we order Thai rice or Basmati Rice from an online shop."* (Interview #16, Male, Sri Lanka)<br>*"Natto is super nice. I have a sensitive stomach, and I think Natto has helped me a lot."* (Interview #18, Male, Mexico)
	Post-migration lifestyle	**1. Time restrictions and busy lifestyle**<br>***Preference of simple food preparation methods that does not take much time**<br>*"Now I eat kind of Japanese style or sometimes in Peruvian style. But very simple, I like things that does not take much time. Peruvian food is too much complicated. Takes time."* (Interview #3, Female, Peru)<br><br>***Bulk food preparation and storing, choosing foods that do not spoil quickly**<br>*"For me, the main reason is the lack of time. Meat, I prepare one time a week, it is okay. Because I can prepare on the weekend. But vegetables… it is not long-lasting. So, if I prepare once, it can last for 2 or 3 days only. So, I cannot prepare much vegetables at once. I do not have enough time to prepare vegetables many times. Also, fruits, I cannot buy too much at once and I cannot shop many times. So, the amount of fruits and vegetables I eat is less than in Myanmar".* (Interview #9, Female, Myanmar)<br><br>***Missing meals**<br>*"Here, I don't have much time because of the work. So, I usually miss my breakfast and morning tea here in Japan."* (Interview #15, Male, Sri Lanka)<br><br>**2. Contrast between pre- and post-migration lifestyles**<br>*"When I moved to Niigata it was the first time I have ever lived in one place and had a full Kitchen to cook so I enjoy preparing and cooking my favorite dishes from my travels around the world. When you actually have a kitchen to cook, you take the advantage of it."* (Interview #5, Male, USA)
Socio-cultural	Contrast	**1. Influence of the characteristic features of Japanese food culture: raw fish, variety of root vegetables, one**

factors	between traditional and Japanese food culture	**bowl meals, incorporating tea with meals**<br>*"I started to eat more raw fish because it is the culture of Japan. In Russia, we originally do not eat raw fish, only in a sushi restaurant or something. Another big difference compared to Russia in Japan is Roots. In Russia, we do not eat roots that much. We eat ordinary potatoes a lot, yes. But ordinary potato. Not sweet potato. Not Satoimo (Taro roots)."* (Interview #4, Female, Russia)<br>*"Back home, when I have my lunch, I have one bowl of soup, one bowl of my main dish, vegetables, one bowl of rice or pasta, like three. But here, I only get one bowl, with everything inside, like soup, noodle or rice, and vegetables, in the same one."* (Interview #14, Female, Türkiye)<br>*"Because of the weather and also the Japanese culture, incorporating tea with every meal has been another change that happened."* (Interview #20, Female, Philippines)<br><br>**2. Positive effects of the Japanese food culture (light meals, fresh food, healthy foods, comparatively low temptations)**<br>*"In Japan we eat the right foods and I eat lots of meat. I think its little bit harder to maintain a good diet in America because the temptations are stronger like sweets better and different varieties of foods."* (Interview #11, Male, USA)<br>*"I think that Japanese people try to eat fresh or raw food as much as possible, for example, they use fresh vegetables for salads, and they eat raw fish as sushi, so I think they try to eat raw food than Sri Lankans. I mean in Sri Lanka sometimes we stir fry the green leaves with a bit of oil without eating it raw."* (Interview #16, Male, Sri Lanka)<br>*"In my country, you have the temptation to eat oily and spicy foods as there are many street foods like Biriyani which are not healthy…..because those are not a balanced diet as they don't represent all food categories. Even though you try to avoid you will find it difficult, because people use it and you get tempted. Here, there is no temptation so, I can eat healthily."* (Interview #8, Female, Mauritius)<br><br>**3. Problems encountered with Japanese food culture (frequent meals, lack of vegan and vegetarian options, additives)**<br>*"After a Japanese meal, I am getting hungry faster than in Kazakhstan. For example, after my lunch [in Kazakhstan], I don't want to eat like for 5 or 7 hours. I don't think about food. Here [in Japan], it's the same size [the meal size], but they use quite light food like salads and fish. So, after 2-3 hours, I get hungry.* (Interview #19, Female, Kazakhstan)<br>*"It is very difficult to be vegan in Japan."* (Survey open comments, Male, Australia)<br>*"Although the diet is a lot more varied in Japan, which is good, it is carb-heavy and contains lots of additives. I eat far too much sodium after moving to Japan, and also sugar is in everything, a lot more than in Western food and cooking. I feel it is easier to eat more healthily in my home country."* (Survey open comments, Female, Australia)
	Relationships with Japanese	**1. Broadened view of Japanese traditional food culture: experience and learn about authentic tastes, ingredients, preparation methods**

	people	*"I have a Japanese friend who is quite close to me. Since we share some time together, and because he is very good at cooking, I learned the original taste of Japanese food and how to appreciate the ingredients, combining them properly for different types of foods. Because of him, I can enjoy eating natto, miso soup, and boiled foods in Japanese taste."* (Interview #3, Female, Peru)<br>*"I used to live with these two Japanese, and one guy eats super nice, he has a lot of tricks of Japanese cuisine in general, and once he said like 'I am making my own soy milk,' and then I asked 'how?,' and he was like 'I just put some tofu in the blender, and that's it,' I was like 'hmm, It might work.' So, now I eat it like almost every day. At least, five days a week."* (Interview #18, Male, Mexico)<br><br>**2. The effect of Covid-19 pandemic**<br>*"Before the pandemic, I used to spend time with my Ikebana (Japanese art of flower arrangement) group; for example, we wear Kimono then go out and eat together some Japanese foods. But, now I don't."* (Interview #1, Female, Chile)
	Religious influence	**Religion-associated food restrictions**<br>*"I eat outside very rarely because of my diet restrictions like.. I don't eat pork. So, I have to check before eating and it is very inconvenient. So, I usually cook."* (Interview #8, Female, Mauritius)<br><br>*"Because I am related to Islam, I don't eat beef unless it's like halal and pork. Also, some foods that include alcohol or wine to make it tastier. So, when I buy food, I look in foods whether they have those materials inside."* (Interview #14, Female, Türkiye)

Table S2.2: Factors influencing changes in PA since migration

Factors	Subthemes (bold) and Illustrative Quotes (italics)
Climate	**Cold winter with heavy snowfall in Niigata limits engaging in outdoor PA or opting for indoor PA**<br>*"The weather in Niigata is cold, so in winter, I avoid going outside much. So, I walk less, much less, in winter."* (Interview #1, Female, Chile)<br>*"I used to work out all the time and used to be at the gym all the time. I tried to run outside and do more exercise here in Japan but the weather is much colder here than in California. California area where I used to live, we don't get snow, so we don't have the long winters and have more time to go outside. I can still run in the snow when it's not too bad. But I probably did a little bit more exercise when I lived in America than in Japan. I've been trying to do less cardio stuff and more weight-lifting stuff because it's so cold, so I do like push-ups and set-ups."* (Interview #11, Male, USA)

Post-migration lifestyle	**1. Changes in social life compared to home country**<br>*"My social life is like almost nothing compared to Chile. I used to go out with my friends a lot back home. On those occasions, we used to dance, for example, so I feel like I am moving less here in Japan. Especially, now I feel like, I am bigger than before. Because I walk less and don't go out with my friends as before."* (Interview #1, Female, Chile)<br><br>**2. Changes in work environment**<br>*"Exercise is, actually in Myanmar also I didn't do. I didn't exercise. But how can I say, in Myanmar, my work is in the clinic, so even though I didn't do any exercise I need to walk. So in the daytime, during office hours, I am mostly walking. But in Japan, most of my time is spent on research and writing papers, most of the work is office work. That's why, my daily life becomes less moving and mostly sitting in the university. It is kind of job-specific."* (Interview #9, Female, Myanmar)
Characteristics of Japanese culture	**1. Health-conscious lifestyle in Japan (being in good shape, riding bicycles, jogging) compared to the home country**<br>*"Most people you meet in your daily life here are in good shape. Compared to other countries, I see more people out jogging daily and the gym I go to is always busy. I find that inspiring. I would say that a higher percentage of people here are more health-conscious than in my home country."* (Interview #5, Male, USA)<br>*"As for the physical activity there is a major change because I never did physical activity in my country. Because I'm used to driving cars [in Indonesia]. But here, I have to walk or I have to ride a bike if I want to go somewhere, So it's a quite major change for me."* (Interview #17, Female, Indonesia)<br><br>**2. Perceived safety to engage in outdoor PA**<br>*"In terms of exercise, I would say that living in Japan helped my overall health since I would usually walk around 10,000 steps or more per day. The safety also adds up to everything. I can go out even at night without any problem."* (Interview #20, Female, Philippines)<br><br>**3. Small apartment rooms limits movements**<br>*"Here, I am too much at home and don't move. And my apartment is small. So, when I am at home I walk less, so I think my weight is increasing."* (Interview #1, Female, Chile)
