# Supplementary material for: Post-Migration Changes in Dietary Patterns and Physical Activity among Adult Foreign Residents in Niigata Prefecture, Japan: A Mixed-Methods Study
Source: Nutrients. 2023 Aug 19;15(16):3639. doi: 10.3390/nu15163639 (PMC10458835; doi:10.3390/nu15163639)
Supplement: Supplementary file 1 [file nutrients-15-03639-s001.zip › Supplementary File S3.pdf]

Figure S1: Thematic Map: Environmental factors influencing post-migration dietary changes

Factors	Why?	How?	Associated factors
Affordability of foods and ingredients	1. Higher prices of some foods and ingredients<br>2. Considerably lower prices of Japanese local foods<br>3. Cost of eating outside	1. Opt for cheaper foods<br>2. Buying smaller quantities<br>3. Purchase only when the prices are lower<br>4. Start to prepare own meals	Duration of stay<br>Employment status
Availability and accessibility of foods and ingredients	1. Less/ non-availability of foods and ingredients<br>2. Difficulty to procure in the same quantities or qualities as in the home country<br>3. Limited number of foreign food restaurants<br>4. Challenges in accessing traditional foods and ingredients<br>5. Wide availability and easy accessibility to Japanese local foods	1. Use of substitute ingredients for unavailable traditional ingredients<br>2. Fusion of Japanese and traditional cuisines<br>3. Ignoring the non-availability of ingredients that do not affect the flavour of traditional dishes significantly<br>4. Giving up the preparation of some traditional dishes<br>4. Driving to other cities where international stores are available<br>5. Ordering online<br>6. Acquiring the skills to prepare and cook traditional/ international dishes independently	Duration of stay<br>Language proficiency
Food safety and related information	1. Perceived safety/ unsafety about foods and ingredients in Japan<br>2. Lack of food-related information or knowledge	1. Buying food without the fear of expiration or cleanliness<br>2. Reluctant to buy products without detailed information<br>3. Not preparing foods that are unavailable in the home country without related information on preparation methods	
Climate	Cold weather in Niigata	Changes in food intake and preparation methods	

Figure S2: Thematic Map: Individual factors influencing post-migration dietary changes

Factors	Why?	How?
Family structure/ living status in Japan	1. The burden of preparing complicated or time-consuming meals for one person<br>2. Japanese-foreign households<br>3. The newfound responsibility of procuring and preparing foods<br>4. Changes in meal-associated family traditions	1. Opt for simple and non-complicated meals<br>2. Bi-cultural meal patterns (traditional cuisines of the foreign resident, and Japanese cuisines)<br>3. Start to procure and prepare own food<br>4. Not following meal-associated practices that enjoyed with the family at the home country such as tea time
Food preferences and limitations	1. Preference for traditional foods/ tastes<br>2. Newfound preference for Japanese foods/ tastes<br>3. Willingness to try new foods<br>4. Ingrained habits and beliefs<br>5. Health-related factors	1. Avoiding Japanese products with flavors different from traditional products<br>2. Choosing foods with similar tastes or qualities to traditional foods<br>3. Increased intake of Japanese food associated with newfound preference for Japanese foods/ tastes, health related factors<br>4. Limited intake of Japanese foods due to taste preferences, health-related factors or ingrained beliefs<br>5. Fusion of Japanese and traditional cuisines
Post-migration lifestyle	1. Time restrictions and busy lifestyle<br>2. Contrast between pre- and post-migration lifestyles	1. Opting for simple foods that do not require much preparation time<br>2. Bulk food preparation and storing<br>3. Choosing foods that do not spoil quickly<br>4. Fusion of traditional and Japanese cuisines<br>5. Missing meals (breakfast)<br>6. Start to prepare and procure own food

Figure S3: Thematic Map: Socio-cultural factors Influencing post-migration dietary changes

Factors	Why?	How?	Associated factors
Contrast between traditional and Japanese food culture	1. Influence of the characteristic features of Japanese food culture<br>2. Positive effects of Japanese food culture<br>3. Problems encountered with Japanese food culture<br>4. Notions regarding the Japanese (food) culture	1. Incorporating characteristic features of Japanese food culture into their diet<br>2. Eating healthier/ unhealthier than in the home country<br>3. Frequent meals<br>4. Pre-migration changes in the diet in preparation for adapting to Japanese (food) culture	
Relationships with Japanese people	1. Broadened view of Japanese traditional food culture<br>2. The effect of COVID-19 pandemic	1. Experience and learn about authentic tastes, ingredients, preparation methods<br>2. Limited opportunities to eat together	Language proficiency
Religious influence	Religion-associated food restrictions	1. Checking the ingredients of foods before purchasing<br>2. Preparing own meals<br>3. Limit eating out	

Figure S4: Thematic Map: Factors influencing post-migration PA changes

Factors	Why?	How?
Climate	1. Cold winter and heavy snowfall in Niigata	1. Reduced outdoor PA (going out, cardio workouts) in winter<br>2. Opt for indoor PA (ex: weight-lifting, push-ups) in winter
Post-migration lifestyle	1. Changes in social life compared to the home country<br>2. Changes in work environment	1. Going out less often than in home country<br>2. Sedentary lifestyle associated with the type of work
Japanese culture	1. Health-conscious lifestyle in Japan<br>2. Perceived safety<br>3. Small apartment rooms	1. Engage in PA (riding bicycles, jogging)<br>2. Going out even at night<br>3. Limited movements inside the home
